# Genetic selection for reduced health treatment costs in Holstein cows: implications from a long-term study

**DOI:** 10.3389/fgene.2023.1254183

**Published:** 2023-09-22

**Authors:** Michael R. Donnelly, Amy R. Hazel, Leslie B. Hansen, Bradley J. Heins

**Affiliations:** Department of Animal Science, University of Minnesota, St. Paul, MN, United States

**Keywords:** health cost, Holstein, dairy cattle, conformation, type traits

## Abstract

The objective of this study was to estimate genetic parameters of health treatment cost of Holstein cows from producer-recorded health treatments in 8 herds over an 8-yr period of time. Genetic parameters of health treatment cost were estimated in first (*n* = 2,214), second (*n* = 1,487) and third (*n* = 800) parities of US Holstein cows. The health treatments were uniformly defined and consistently recorded by 8 high-performance dairy herds in Minnesota from 2008 to 2015. A fixed treatment cost was assigned to 14 types of health treatments, and the cost included the mean veterinary expense obtained from the veterinary clinics that serviced the 8 herds, pharmaceuticals, and labor cost. The labor cost was $18/h, and the time incurred for each type of health treatment was determined from interviews with the herd owners. The 14 types of health treatment costs were partitioned into 5 categories: mastitis (including mastitis diagnostic test), reproduction (cystic ovary, retained placenta, and metritis), lameness (hoof treatments), metabolic (milk fever, displaced abomasum, ketosis, and digestive), and miscellaneous (respiratory, injury, and other). Health treatment cost for each cow was summed by category within lactation and also across categories within lactation. The estimates of heritability for health treatment cost were 0.13, 0.04, 0.10, 0.12, and 0.04 for the mastitis, reproduction, lameness, metabolic, and miscellaneous categories, respectively, in first parity. Genetic correlations between categories of health treatment cost in first parity were greatest for mastitis and reproduction (*r* = 0.85); however, phenotypic correlations between all categories were small (*r* < 0.16). Total health treatment cost had a large genetic correlation with somatic cell score (0.93) and 305-d milk production (0.44) in first parity; however, the genetic correlation (−0.60) between total health treatment cost and udder depth in first parity indicated a genetic relationship exists between shallow udders and less total health treatment cost. Total health treatment cost across categories had a heritability estimate of 0.25 in first parity, 0.16 in second parity, and 0.17 in third parity. Consequently, genetic selection for reduced health treatment cost should be possible by using producer-recorded health treatment records supplemented with treatment costs.

## 1 Introduction

The long-term genetic improvement of production and conformation traits in the Holstein breed has negatively affected the health and welfare of dairy cows (Oltenacu and Broom, 2010), and the decreased health of cows usually reduces profitability. The total economic cost of health disorders are often not visualized by dairy producers, but those costs may substantially erode profit per cow for diseased animals. For example, Guard (2008) reported the total cost of a displaced abomasum was $494 and the cost of each case of lameness was $469. Also, health disorders impact the profitability of cows through higher involuntary culling, loss of cull cow income from death, decreased milk production, and more milk withholding (Zwald et al., 2004a). Furthermore, the impaired health of cows leads to reduced fertility (Weigel, 2004), and each additional day open (days from calving to conception) results in a reduction of $2.75 in profit (VanRaden and Cole, 2014). However, genetic improvement of health traits could enhance the longevity of dairy cattle (Buonaiuto et al., 2023). Consequently, selection for cow health is of growing interest to dairy producers.

Direct selection against health disorders has been successfully utilized in Scandinavian countries for more than 30 yr (Philipsson and Lindhé, 2003), because only veterinarians are permitted to treat health disorders and they uniformly record all health treatments for cows in a national database. However, in many countries (including the United States), the recording of health treatments are voluntary rather than mandatory, and producer-recorded health data is often variable and incomplete. Low-quality data for health treatments may lack utility for both herd management and for genetic evaluation.

Estimates of heritability for health traits of dairy cows have generally been low; however, the highest estimates have been reported for the health disorders that are most frequently recorded due to either their high cost or their ease of recording (Zwald et al., 2004a; Appuhamy et al., 2009; Vukasinovic et al., 2017). For example, the estimates of heritability for displaced abomasum (from 0.03 to 0.21) and mastitis (from 0.01 to 0.09) make these particular disorders attractive candidates for genetic selection. Despite the typically low estimates of heritability, significant genetic correlations between health disorders have been reported (Koeck et al., 2012), including 0.44 for displaced abomasum with metritis and 0.49 for mastitis with lameness. Furthermore, Rupp and Boichard (1999) found an antagonistic genetic correlation (0.45) between milk production and clinical mastitis. Mounting evidence has documented that selection for increased body size and lower body condition of Holstein cows has been detrimental to health of cows. In particular, a long-term selection study on body size found large Holstein cows had 30% greater cost of healthcare than small cows (Becker et al., 2012). Furthermore, Dechow et al. (2004) estimated dairy form, which is a measure of angularity, was highly correlated (0.85) with a composite of all health disorders from national United States data.

Perhaps, health disorders were slow to be included in official genetic evaluation in the United States because traits with low incidence rates and those recorded in a binary fashion present challenges for effective selection (Zwald et al., 2004a). Moreover, Parker Gaddis et al. (2012) and Neuenschwander et al. (2012) suggested producer-recorded health data require large numbers of cows over multiple years in order to detect reliable differences between sires within breeds. Advances in genomic technologies may assist in farmers selecting for health traits in the future. Selection for health treatment costs may greatly benefit from genomic technology implementation (Gaddis et al., 2020; Brito et al., 2021; Jones and Wilson, 2022). The objective of this study was to estimate genetic parameters of health treatment cost (as opposed to binary incidence rates) of Holstein cows from producer-recorded health treatments that were uniformly defined and consistently recorded in 8 herds over an 8-yr period of time.

## 2 Materials and methods

### 2.1 Experimental design

A long-term study of Holstein cows in 8 high-performance herds in Minnesota was initiated by the University of Minnesota in 2008. The weighted mean production for all cows in the 8 herds was 14,019 kg of milk, 519 kg of fat, 433 kg of protein, and the herds ranged in size from 302 to 1,932 cows with a mean herd size of 981 cows; however, some cows in the herds were not Holstein. All cows in each of the 8 herds were housed in a 4- or 6- row freestall facility and fed a total mixed ration during lactation.

Only proven AI bulls with very high rank for Net Merit (VanRaden and Cole, 2014) were chosen by the herd owners in consultation with 2 genetic advisors employed by Minnesota Select Sires Co-op, Inc., St. Cloud, MN. Herd owners were asked to select proven AI bulls that ranked among the top 10% of available bulls for the Net Merit index within the Holstein breed. Cows were correctively mated by the 2 genetic advisors on an individual basis for conformation and heifers were correctively mated on the conformation scores of their dam when possible. Also, inbreeding protection was provided for all matings of cows and heifers.

### 2.2 Experimental units

The first 3 lactations of 4,894 Holstein cows across the 8 herds were considered for the study. Cows initiated a first lactation from March 2008 to October 2015 and were required to calve for a first time during this time span in order to contribute data in second and third parity; however, data for this study is censored because 287 cows had not completed first, 218 cows had not completed second, and 109 cows had not completed third parity by the end of the study (November 2015). Lactations of cows commenced with an abortion (*n* = 267) were removed from the analysis. Also, the lactations of cows that calved during the final month of the study (*n* = 77) did not have the opportunity to accumulate 30 d of health treatment cost and were removed from the data. Consequently, mean lactation length was 332 ± 2.7 d.

Age at first calving had a mean of 24.0 months and ranged from 20 to 36.6 months for first-lactation cows. Age at calving was 36.8 months for second lactation cows and 49.5 months for third lactation cows. Mean days open was 127 days for first-lactation cows, 140 days for second lactation cows, and 147 days for third lactation cows.

All cows and their dams were required to be sired by a Holstein AI bull that had a sire code assigned by the National Association of Animal Breeders (Columbia, MO). In order to validate the sire and maternal grandsire identification, AI bulls were also required to have at least 2 descendants (either daughters or granddaughters) in the data. Following all edits, 2,214 cows sired by 260 AI bulls remained for analysis and a total of 4,501 lactations were analyzed. The distribution of cows by herd and parity is in Table 1.

**TABLE 1:** Numbers of cows analyzed for cost of health treatments by herd and parity.

	Parity		
Herd	1	2	3
A	394	246	125
B	227	157	100
C	427	299	181
D	402	279	150
E	179	117	59
F	152	105	48
G	250	159	66
H	183	125	71
Total	2,214	1,487	800
Percentage of total by parity (%)	49	33	18

### 2.3 Trait descriptions

The treatment of 14 individual health disorders (Table 2) were uniformly defined and consistently recorded across the 8 herds with Dairy Comp 305 (Valley Ag Software, Tulare, CA). To distinguish between multiple treatments of the same illness event during the lactation of each cow, a new health treatment observation was assigned only if 3 or more days elapsed between hoof treatments, if 5 or more days elapsed between digestive, ketosis, mastitis, metritis, milk fever, and respiratory treatments, or if 7 or more days elapsed between cystic ovary treatments. Only a single treatment observation was permitted per lactation of a cow for displaced abomasum, retained placenta, and miscellaneous reproduction. No restriction on days between treatments was applied to mastitis diagnostic test, injury, or other treatments; however, only 1 treatment per day was permitted. The health treatments were assigned to 1 of 5 categories: mastitis (MAST), reproduction (REPRO), lameness (LAME), metabolic (META), and miscellaneous (MISC), which are itemized in Table 2.

**TABLE 2:** Specific health treatments included in the health treatment categories.

Category	Abbreviation	Treatment
Mastitis	MAST	Mastitis
		Mastitis diagnostic test^a^
Lameness	LAME	Hoof treatment^b^
Reproduction	REPRO	Cystic ovaries
		Retained placenta
		Metritis
		Miscellaneous reproduction^c^
Metabolic	META	Milk fever
		Displaced abomasum
		Ketosis
		Digestive^d^
Miscellaneous	MISC	Respiratory
		Injury
		Other treatments

^a^ Mastitis diagnostic test included milk culture and California Mastitis Test (Immucell, Portland, ME).

^b^ Hoof treatment included dermatitis, infectious pododermatitis, foot ulcer, and other hoof treatments.

^c^ Miscellaneous reproduction included abortion treatments, caesarean section, pyometria, uterine disorders (adhesion, mass, prolapse, and torsion), and mummified calf.

^d^ Digestive included *clostridium*, traumatic reticuloperitonitis, hemorrhagic bowel syndrome, peritonitis, twisted cecum, lack of appetite, or any other digestive treatment.

The costs of health treatments were completed during February 2016 by the first author and was exclusive to the 8 herds in this study (Donnelly et al., 2023). Health treatment costs were based on interviews with the dairy farmers and their herd veterinarian. The veterinary cost for each health treatment was obtained from the veterinarian providing service to each of the 8 herds. Pharmaceutical expenses were sourced either directly from the herd veterinarians or based on the average catalog prices of pharmaceuticals provided by 5 veterinary service vendors serving Minnesota. Veterinary cost included veterinarian labor, supplies, and pharmaceuticals used for each specific health treatment. Labor cost included the time required by herd owners for segregation, restraint, and therapy and was assigned a value of $18/h. The hourly rate and the time attributed to each type of health treatment was the mean rate and time reported during interviews of the 8 herd owners. A fixed cost was calculated for each of the 14 types of health treatments, and cost was the sum of veterinary expense, pharmaceuticals, and labor cost associated with each specific type of treatment.

The observations for health treatment cost of each cow were summed within each of the 5 health categories by parity (including the subsequent dry period) to obtain a lactational cost for MAST, REPRO, LAME, META, and MISC. Likewise, the cost of 4 specific health treatments (displaced abomasum, ketosis, metritis, and retained placenta) were summed by treatment type and by parity for each cow. Finally, health treatment cost across all 14 treatment types was summed to obtain the total health cost (THC) by parity for individual cows. For cows that left the herd during lactation, THC was simply the sum of health costs from calving until the day of disposal and no adjustment was made for DIM at disposal. Likewise, for cows with records in progress at the end of the study (*n* = 614), THC for that parity was the sum of health costs incurred from calving until the end of the study.

Best Prediction (Cole and VanRaden, 2009), which is routinely used for genetic evaluation in the United States, was applied to individual test-day observations to calculate the actual 305-d milk, fat, and protein production (not mature equivalent production) as well as SCS of cows for their first 3 lactations. Five of the 8 herds had monthly test-day observations, and the other 3 herds had test-day observations at least 8 times per yr. Test-days were required to be at least 4 DIM and milk weights were required to be greater than 2.27 kg, fat percentage was required to be at least 1.0% but no greater than 9.9%, and protein percentage was required to be at least 1.0% but no greater than 6.0%. Best Prediction adjusted records for age at first calving and projected records to 305 d for records less than 305 d. Some cows left herds prior to a first test day; therefore, a total of 2,155 first parity, 1,466 second parity, and 757 third parity records were available for analysis.

Conformation of cows was scored once during first lactation by 1 of 2 evaluators employed by Minnesota Select Sires Co-op, Inc., St. Cloud, MN. Three conformation traits (stature, dairy form, and udder depth) were subjectively scored on a 1-to-9 linear scale and a score of 5 was considered the biological mid-point for each trait (Select Sires, Inc., Plain City, OH). Most cows were scored in early lactation (32 ± 0.3 DIM). Stature was scored, but not measured, at the withers and each score increment represented approximately 2.54 cm of height. Cows with a score of 1 were less than 130 cm and cows with a score of 9 were greater than 150 cm. For dairy form, a score of 1 represented heavy, coarse-boned cows that lacked openness of rib, whereas 9 represented clean, open-ribbed, long-necked cows. Udder depth described the position of the udder floor relative to the hocks and each score increment represented approximately 2.54 cm. Cows with a score of 1 had udder depth at least 5 cm below the point of the hock, and cows with a score of 9 had udder depth at least 15 cm above the point of the hock. Some cows either left the herds prior to scoring or were not scored; therefore, conformation was analyzed for 2,090 first-parity cows.

### 2.4 Genetic analysis

Linear animal mixed models were fitted using restricted maximum likelihood (ASReml; Gilmour et al., 2015). Pedigrees of the Holstein cows were provided by the Council on Dairy Cattle Breeding (Bowie, MD); however, only relationships among sires and maternal grandsires of cows were retained for analysis. All models included the fixed effect of herd and cow nested within herd was a random variable. A preliminary analysis examined the fixed effects of year and season of calving; however, neither effect significantly accounted for variation of the dependent variables.

The following model was used for analysis:

Yijk=μ+Hi+Cj+εijk

where *Yijk* are the observed values, *μ* is the overall population mean, *Hi* is the fixed effect of *i* ^*th*^ herd, *Cj* is cow within *i* ^*th*^ herd as a random effect, and *eijk* is the residual error with ε ∼ *N*(0,σ^2^_e_). Cow was distributed following *N*(0,Aσ^2^_a_), where A is the relationship matrix based on the pedigree and σ^2^_a_ is the genetic variance.

Three distinct statistical models were used for analysis. First, a univariate linear model was fitted to obtain least squares means, estimates of heritability, and standard errors for health treatment cost for each of the 5 health treatment categories, THC, the 4 specific health treatment costs, and the 3 conformation traits in first parity. The second was a bivariate linear model, which was fitted to obtain pairwise genetic and phenotypic correlations between the 5 categories of health treatment cost, THC, 4 specific health treatment costs, 305-d production, and conformation in only first parity. Correlations were obtained in a pairwise manner because convergence of a multitrait model including all dependent variables simultaneously was not feasible with ASReml. Lastly, a multivariate linear model was fitted to obtain least squares means, estimates of heritability, and standard errors for THC, 305-d production, and SCS during first, second, and third parity. For this model, each of the dependent variables were analyzed separately and each of the 3 parities were defined as 3 distinct traits in the multivariate model.

## 3 Results and discussion

### 3.1 Least squares means

The mean cost for 5 categories of health treatment, THC, and mean cost for 4 specific health treatments (Table 3), as well as frequencies of health treatments were generated by the univariate analysis that included only a single parity of Holstein cows. The REPRO had the highest percentage contribution (28%) to THC and a first-parity cost of $15.28. The cost of REPRO mostly reflected treatment cost for metritis, which had a cost of $9.95. Also, LAME (23%) and MAST (20%) contributed substantially to THC with costs of $12.89 and $10.88, respectively. Donnelly et al. (2023) analyzed the costs of health treatments subdivided by 6 intervals of lactation, and found the treatment costs for REPRO, META, and MISC were more concentrated during early lactation, while treatment costs for MAST and LAME were distributed throughout first lactation. In that study, 41% of THC during first parity occurred during the first 30 d of lactation (Donnelly et al., 2023).

**TABLE 3:** Least squares means, standard errors, percent of total health cost (THC) for the treatment costs of 5 health categories, THC, 4 specific health treatments, frequencies of health treatments, and the average conformation scores (1–9 scale) in first parity from univariate analysis.

Trait	LSM	SEM	Percentage of total	Frequency of treatment
Category^a^	------------($)------------		(%)	(%)
MAST	10.88	2.36	20	26.5
REPRO	15.28	4.54	28	13.3
LAME	12.89	2.13	23	26.7
META	8.02	3.82	15	7.7
MISC	8.13	2.28	15	14.1
THC	55.18	7.89	100	
Specific health treatment				
Metritis	9.95	3.53	18	8.2
Retained placenta	2.12	1.22	4	2.7
Displaced abomasum	4.91	3.33	9	1.7
Ketosis	0.60	0.42	1	1.5
Conformation	---------Score---------			
Stature	5.6	0.14	--	--
Dairy form	5.4	0.16	--	--
Udder depth	6.7	0.13	--	--

^a^ MAST, mastitis; REPRO, reproduction; LAME, lameness, META, metabolic; MISC, miscellaneous.

The frequencies of health treatments ranged from 7.7% for META to greater than 26% for both MAST and LAME. For specific treatments, displace abomasum and ketosis were 1.7% and 1.5%, respectively, and metritis was higher (8.2%). Parker Gaddis et al. (2020) reported an incidence rate of 1.6% for displaced abomasum, which was similar to the current study. However, incidence of ketosis was lower, and incidence of mastitis and metritis were higher in the current study compared to health events in the 2019 national dairy database (Parker Gaddis et al., 2020).

Parker Gaddis et al. (2012), Becker et al. (2012), and Hardie et al. (2022) reported lactational incidence rates were highest for mastitis and lameness, which was similar to the findings of the current study.

The means for the 3 conformation traits in first parity were as expected for young Holstein cows in early lactation (Table 3). The score of 5.6 for stature (on a 9-point scale) converts to approximately 141 cm of stature at the withers (Select Sires, Inc., Plain City, OH). For dairy form, the least squares mean score of 5.4 indicated these first-parity cows in early lactation were slightly more angular than the biological midpoint for dairy form. First-parity cows in this study had mean udder depth of 6.7, which indicated that the average cow had an udder floor approximately 9 cm above the point of hock. Shallow udders in first-parity Holsteins were also reported in a French study by Rupp and Boichard (1999) and were also scored on a similar 9-point scale with an average score of 6.3 for udder depth.

The multivariate analysis was used to fit means for THC across the first 3 parities (Table 4), and the mean THC in first parity from that analysis differed by only $2.73 from the univariate analysis. The least squares means for THC from the multivariate analysis increased with parity (Table 4) and ranged from $57.91 in first parity to $87.95 in third parity. The THC observed in first parity in this study may not seem expensive on a per-cow basis; however, the average herd in this study calved 444 first-parity cows during 2015, which converts to at least $25,000 annually in THC. Furthermore, the 8 herds had more multiparous than primiparous cows; therefore, the economic impact of THC for these 8 herds was substantial and may greatly impact profitability.

**TABLE 4:** Least squares means and pooled SEM for total health treatment cost (THC), 305-d production, and SCS in parities 1 to 3 from multivariate analysis across parities.

	Parity			
Trait	1	2	3	Pooled SEM
THC ($)	57.91	73.92	87.95	13.95
Milk (kg)	10,943	12,628	13,018	261
Fat (kg)	395	449	464	9.5
Protein (kg)	331	389	401	7.1
Fat + Protein (kg)	726	838	865	15.8
SCS	2.21	2.33	2.63	0.13

The least squares means for production traits in parities 1 to 3 (Table 4) were also calculated from the multivariate analysis, and the means fit expectations for the high performance of these 8 herds. The least squares means for milk, fat, protein, and fat plus protein production increased (*p* < 0.05) with parity, and the milk production of cows in this study was far superior to the average fluid milk production (10,157 kg) of US cows enrolled in milk recording during 2015 (US Department of Agriculture, 2016). Furthermore, the means of SCS were not greater than the average SCS in first (2.0), second (2.0), and third and greater (2.6) parity of US Holstein herds enrolled in milk recording (Dairy Records Management System, 2023).

### 3.2 Estimates of heritability for health treatment cost in first parity

Heritability was estimated for the 5 categories of health treatment cost and also for THC with a univariate analysis that only considered first parity of cows and is in Table 5. The estimate of heritability (0.13) for cost of MAST was higher than the estimates of heritability of 0.06 and 0.09 reported by Gernand et al. (2012) and Zwald et al. (2004a), respectively. The estimate of heritability for cost of MAST in this study supports the suggestion of Nash et al. (2000) and Zwald et al. (2004a) to include both mastitis and SCS in a selection index to improve the effectiveness of selection for mastitis resistance.

**TABLE 5:** Estimates of heritability (in bold on the diagonal, with SE in parentheses) from the univariate analysis, and genetic correlations (above the diagonal, with SE in parentheses) and phenotypic correlations (below the diagonal, with SE in parentheses) from pairwise bivariate analysis for the treatment costs of 5 health categories^a^ and total health treatment cost (THC) in first parity.

	MAST	REPRO	LAME	META	MISC	THC
MAST	**0.13** ^b^ (0.05)	0.85^b^ (0.20)	0.34 (0.28)	0.52 (0.27)	0.66 (0.34)	0.92^b^ (0.10)
REPRO	0.00 (0.02)	**0.04** (0.03)	0.41 (0.35)	0.73^b^ (0.29)	0.59 (0.40)	0.91^b^ (0.09)
LAME	0.03 (0.02)	−0.01 (0.02)	**0.10** ^b^ (0.04)	0.56^b^ (0.25)	0.21 (0.38)	0.65^b^ (0.18)
META	0.02 (0.02)	0.14^b^ (0.02)	0.02 (0.02)	**0.12** ^b^ (0.05)	0.40 (0.37)	0.85^b^ (0.10)
MISC	0.04^b^ (0.02)	0.02 (0.02)	−0.05^b^ (0.02)	0.16^b^ (0.02)	**0.04** (0.03)	0.72^b^ (0.20)
THC	0.34^b^ (0.02)	0.66^b^ (0.01)	0.27^b^ (0.02)	0.63^b^ (0.01)	0.39^b^ (0.02)	0.27^b^ (0.07)

^a^ MAST, mastitis; REPRO, reproduction; LAME, lameness; META, metabolic; MISC, miscellaneous.

^b^ Estimate was significantly different from zero based on 95% CI.

The estimate of heritability (0.04) for cost of REPRO in first parity (Table 5) was low and not significantly different from zero. Previous studies have reported similarly low estimates of heritability (0.02) for the incidence of reproduction disorders across parities (Lyons et al., 1991; Dechow et al., 2004). The health treatment cost for metritis and retained placenta (Table 6) had estimates of heritability of 0.02 and 0.12, respectively, and both of these individual health treatment costs were included for the cost of REPRO. The estimate of heritability of metritis cost in this study was similar to the estimates of heritability (0.01–0.03) from 2 other reports (Van Dorp et al., 1998; Koeck et al., 2012, respectively). However, the estimate of heritability (0.12) for cost of retained placenta in this study was larger than the estimate of heritability (0.07) recently reported by Vukasinovic et al. (2017) from large, producer-recorded data in the United States, and also larger than the 0.04 and 0.03 estimates of heritability found by Gernand et al. (2012) and Koeck et al. (2012), respectively. A possible explanation for the higher heritability for cost of retained placenta in this study may be the clear distinction between treatment of retained placenta and treatment of metritis, because treatments for these two reproductive disorders may have been recorded as a single disorder in other studies of producer-recorded health treatments (Zwald et al., 2004a). Perhaps, the estimates of heritability found in this study suggest the cost of retained placenta is a superior selection criterion to the categorical cost of all REPRO treatments because large variation existed for the cost of metritis and the other costs of specific health treatments summed within REPRO.

**TABLE 6:** Estimates of heritability (in bold on the diagonal, with SE in parentheses) from the univariate analysis, and genetic correlations (above diagonal, with SE in parentheses) and phenotypic correlations (below diagonal, with SE in parentheses) from pairwise bivariate analysis for the cost of 4 specific health treatments in first parity.

	Metritis	Retained placenta	Displaced abomasum	Ketosis
Metritis	**0.02** (0.02)	0.66 (0.48)	0.79^a^ (0.23)	---^b^
Retained placenta	0.21^a^ (0.02)	**0.12** ^a^ (0.05)	−0.37 (0.27)	0.88^a^ (0.20)
Displaced abomasum	0.16^a^ (0.02)	0.01 (0.02)	**0.12** ^a^ (0.05)	0.97^a^ (0.15)
Ketosis	---^b^	0.06^a^ (0.02)	0.24^a^ (0.02)	**0.18** ^a^ (0.07)

^a^ Estimate was significantly different from zero based on 95% CI.

^b^ Convergence was not achieved.

The estimate of heritability of 0.10 for cost of LAME in this study (Table 5) is within the range of estimates (0.02–0.23) for incidence of specific types of hoof health disorders for Holstein cows in Nordic countries (Häggman and Juga, 2013; Ødegård et al., 2013). Hoof health data recorded by professional hoof trimmers in Nordic countries was the foundation for the development of a hoof health selection index, which was integrated into the Nordic total merit index in 2011 (Johansson et al., 2011). The estimate for cost of LAME in this study suggests selection for reduced cost of lameness of dairy cattle is possible in other regions of the world if treatments for LAME are routinely recorded. Selection for reduced lameness may increase profitability of individual cows; however, the consequential improvement of welfare of cows may be even more valuable into the future.

The cost of META had an estimate of heritability of 0.12 in first parity (Table 5), which was higher than the estimate of 0.05 reported by Gernand et al. (2012), but lower than the estimate of 0.17 from Lyons et al. (1991) for incidence of metabolic disorders. Displaced abomasum accounted for 61% of the cost of META (Table 3) and had an estimate of heritability of 0.12 (Table 5) in first parity. The heritability of displaced abomasum cost in the present study was higher than estimates of 0.06 and 0.08 reported for incidence of displaced abomasum by Koeck et al. (2012) and Dechow et al. (2004), respectively. Ketosis also contributed to cost of META cost in this study, and ketosis had an estimate of heritability of 0.18 when evaluated as a specific health treatment (Table 6). Our estimate of heritability for ketosis cost (0.18) was greater than the estimates of 0.01 and 0.09 for incidence of ketosis reported by Appuhamy et al. (2009) and Neuenschwander et al. (2012), respectively. β-hydroxybutyrate is a proxy for ketosis in cows, and heritabilities for β-hydroxybutyrate range from 0.058 to 0.071 (Weigel et al., 2017) and from 0.10 to 0.13 (Lou et al., 2022). However, in Italian Holstein cows, heritability of β-hydroxybutyrate ranged from 0.13 to 0.30 (Benedet et al., 2020). Perhaps, β-hydroxybutyrate measured cow side on farm may provide accurate information for use in genetic evaluation for ketosis. Donnelly et al. (2023) described the substantial treatment costs for specific health disorders (especially displaced abomasum) included in META for this study; however, the incidence of treatments for META is apparently low because the cost of META was only 15% of THC (Table 3). Therefore, selection against META cost may substantially reduce health costs among even a small number of cows with 1 or more metabolic disorders because of the high cost associated with each treatment of META.

The low estimate of heritability (0.04) for MISC cost in first parity was not significantly different from zero and reflected the low genetic control of heath treatments for respiratory, injury, and other treatments. Calculations of the heritability of respiratory disorders are sparse in the literature; however, Lyons et al. (1991) estimated heritability of 0.01 for respiratory disorders. Regarding injury, few injured cows experience treatment because most cows either recover without intervention or exit the herd without treatment when an injury is catastrophic. Therefore, results from this study suggested treatments for injury were highly dependent on environmental factors. Ultimately, the low heritability (0.04) for cost of MISC in this study was anticipated because of the lack of uniform treatment types within the MISC category.

Contrary to a majority of large studies using field data that estimated genetic parameters for incidence of specific health treatments or categories of health disorders in a binary manner (Harder et al., 2006; Koeck et al., 2012; Vukasinovic et al., 2017), this study summed the cost of all disorders for a lactation, which permitted a comprehensive consideration of the genetic control of health disorders. Previous research with binary data typically gave estimates of heritability for health disorders that were less than 0.10; however, the estimate of heritability (0.27) for THC from this study was moderate in first parity (Table 5), despite the lower estimates for each of the 5 treatment categories. The higher estimate of heritability (0.27) found in this study compared with previous research likely resulted from greater variation for THC between cows. Variation in THC in this study may have been caused by the assignment of variable costs for 14 specific health treatments, by permitting treatment incidence of some disorders to be recorded more than once per lactation, or by both. Apparently, sires that transmitted genes for more cost of health disorders to their progeny were more easily identified when cost of treatments were amalgamated compared to analyses in which incidence of health treatments were counted. Few studies have summed incidence or cost of health treatments within a lactation; however, Lyons et al. (1991) estimated a heritability of 0.03 for the sum of all health incidences weighted by their costs and pooled across lactations.

### 3.3 Estimates of heritability for conformation

The heritability estimate (0.42 ± 0.08) for stature was the highest of the 3 conformation traits and was expected because previous studies reported stature had the highest heritability among commonly reported conformation traits (DeGroot et al., 2002; Dechow et al., 2003). The estimate of heritability for dairy form and udder depth in first parity was 0.28 ± 0.06 and 0.32 ± 0.07, respectively. Dechow et al. (2003) reported estimates of heritability of 0.37 for stature and 0.24 for dairy form, which are similar to the estimates of heritability in this study. However, Van Dorp et al. (1998) found a much lower estimate of the heritability for udder depth (0.19). Nonetheless, the heritability estimates from this study are in general agreement with those published by Holstein Association USA (2023), which were 0.42, 0.29, and 0.28 for stature, dairy form, and udder depth, respectively.

### 3.4 Genetic and phenotypic correlations in first parity

Health Categories and THC. Positive genetic correlations were found among all combinations of the 5 categories of health treatment costs in first-parity cows (Table 5). Clearly, genetic predisposition for health treatment cost in one category was accompanied by greater likelihood of health treatment cost in other categories. The genetic correlation (0.85) between the costs of MAST and REPRO was particularly large, and the correlation from this study was greater than the genetic correlations between mastitis and specific types of reproduction disorders reported by Koeck et al. (2012) and Zwald et al. (2004b). Perhaps, the high genetic correlation suggests similar genes control resistance to REPRO disorders and resistance to MAST. The genetic correlation (0.73) between the costs of REPRO and META was also substantial, but higher than the genetic correlation between the incidence of similar types of health disorders of 0.38 reported by Lyons et al. (1991).

Genetic correlations between THC and health treatment cost for each of the 5 categories (Table 5) were all highly positive and ranged from 0.65 (THC with LAME) to 0.92 (THC with MAST). The positive and significant genetic correlations of THC with the health treatment cost of the 5 categories suggest specific bulls may transmit a predisposition for cows to be more prone to many health disorders. Furthermore, the correlations of THC with the cost of REPRO (*r* = 0.91) and THC with the cost of MISC (*r* = 0.72) were not different from unity in first parity because the SE for both correlations were large; therefore, selection for THC may be more effective for lowering the cost of REPRO and MISC than direct selection for only cost of REPRO and MISC, which both had low and non-significant estimates of heritability.

The phenotypic correlations among the 5 categories of health treatment cost were much smaller than the genetic correlations and ranged from −0.05 (MISC with LAME) to 0.16 (MISC with META). Phenotypically, cows with more META cost were more prone to higher costs of REPRO (*r* = 0.14) and MISC (*r* = 0.16). Metabolic disorders, especially ketosis, have been associated with an increase of other infectious diseases and impaired reproduction in other reports (Reist et al., 2003; Walsh et al., 2007); therefore, the significant phenotypic relationship between the costs of REPRO and META in this study was not surprising. Among commercial herds in New York, cows with retained placenta had increased risk of developing mastitis (Schukken et al., 1988). However, our study found no phenotypic correlation (0.00) between the costs of REPRO and MAST in first parity. Phenotypic correlations between THC and the health treatment cost of the 5 categories ranged from 0.27 (LAME) to 0.66 (REPRO). Costs of REPRO and META had the largest phenotypic correlations (0.66 and 0.63, respectively) with THC. The individual treatments with highest cost were found within these 2 categories (Donnelly et al., 2023), which may partially explain their strong phenotypic correlation with the total costs of health treatment in first-parity cows. Furthermore, a moderate phenotypic correlation (0.34) existed between THC and the cost of MAST, which confirmed the result of Hansen et al. (2002), who reported cows with mastitis are more likely to experience other health disorders.

In first parity, the costs of displaced abomasum and ketosis had a genetic correlation (0.97) near unity (Table 6), which indicated genes influencing treatment for displaced abomasum were likely the same genes influencing treatment of ketosis. Similarly, the genes associated with treatment costs of retained placenta and ketosis (*r* = 0.88) may be mostly the same. Also, the genetic correlation (0.79) for costs of metritis and displaced abomasum was likely the explanation for the large genetic correlation (0.73) between the categorical costs of REPRO and META (Table 5).

For the 5 phenotypic correlations estimated among the costs for the specific health treatments (Table 6), 4 of the 5 were significantly greater than zero. Apparently, some cows treated for a first health disorder postpartum may experience a second (or multiple) health disorders postpartum. The phenotypic correlation (0.24) for ketosis and displaced abomasum is comparable to the correlation of 0.27 among the same 2 traits for Holstein cows in a study of Canadian commercial herds (Koeck et al., 2012). A study by LeBlanc et al. (2005) reported postpartum negative energy balance may lead to metabolic disorders, such as ketosis, which is a risk factor for displaced abomasum. The low phenotypic correlations between the cost of specific health treatments in this study agree closely with the correlations between incidences of health disorders from other studies (Van Dorp et al., 1998; Koeck et al., 2012), and this is likely because treatments of retained placenta and displaced abomasum occurred once per lactation and all 4 of these traits were not grouped with other disorders for analysis.

The genetic correlation (0.44) of THC with 305-d milk production was especially unfavorable (Table 7) and provides evidence that the simultaneous selection for lower health cost in conjunction with selection for milk production is very important. Lyons et al. (1991) reported a smaller genetic correlation (0.29) between milk production and the sum of all health incidences. In the current study, fat, protein, and fat plus protein production also had unfavorable genetic correlations with THC, but they were smaller and not significantly different from zero. Perhaps, selection for the solid constituents in milk will not be as detrimental to cow health as the historical selection for fluid milk had been. The genetic correlation (0.93) of THC with SCS was high and other studies have previously documented a large genetic correlation (0.76) of SCS with incidence of mastitis (Täubert et al., 2013). More than likely, the dramatic genetic trend for reduced SCS in the United States since 2001 (Council on Dairy Cattle Breeding, 2023) may have caused a lowering of THC in the Holstein breed during the last 15 yr.

**TABLE 7:** Genetic and phenotypic correlations (SE in parentheses) of total health cost (THC) with 305-d production and SCS in first parity from pairwise bivariate analysis.

	THC	
Trait	Genetic	Phenotypic
Milk	0.44^a^ (0.18)	−0.07^a^ (0.02)
Fat	0.07 (0.21)	−0.08^a^ (0.02)
Protein	0.28 (0.20)	−0.10^a^ (0.02)
Fat + Protein	0.18 (0.21)	−0.09^a^ (0.02)
SCS	0.93^a^ (0.13)	0.14^a^ (0.02)

^a^ Estimate was significantly different from zero based on 95% CI.

Phenotypic correlations between THC and production traits (Table 7) were negative and small. Cows with more health problems and consequently, greater THC, had decreased 305-d production of milk, fat, protein, and fat plus protein. Other studies have also found a slightly negative relationship between health disorders and production (Fourichon et al., 1999; Gernand et al., 2012). A small and positive phenotypic correlation (0.14) in this study between SCS and THC was similar to the 0.22 phenotypic correlation reported by Täubert et al. (2013). Cows with high SCS are expected to have more THC because higher SCS often accompanies greater cost of MAST.

None of the genetic correlations of health treatment cost for the 5 categories, THC, and 4 specific health treatments with stature and dairy form (Table 8) were significantly different from zero because the standard errors were large. These results were contrary to Dechow et al. (2004), who reported a large, antagonistic genetic association of 0.85 between dairy form and all diseases recorded in United States dairy herds. The genetic correlations that were large but non-significant for stature and dairy form with health treatment cost in this study may reflect that cows were scored for stature and dairy form only once in early lactation, and thus had little variation (SEM = 0.13 to 0.16; Table 3); therefore, perhaps, models were unable to detect the underlying true genetic associations. The addition of observations for conformation traits during late lactation and from multiparous cows (e.g., Dechow et al., 2004) would have provided more phenotypic variation to permit elucidation of genetic relationships.

**TABLE 8:** Genetic and phenotypic correlations (SE in parentheses) of conformation^a^ with the treatment costs of 5 health categories, total health cost (THC), and 4 specific health treatments in first parity from pairwise bivariate analysis.

	Stature		Dairy form		Udder depth	
Trait^b^	Genetic	Phenotypic	Genetic	Phenotypic	Genetic	Phenotypic
MAST	−0.22 (0.21)	0.04^c^ (0.02)	−0.30 (0.21)	0.01 (0.02)	−0.84^c^ (0.17)	−0.11^c^ (0.02)
REPRO	0.09 (0.33)	0.01 (0.02)	−0.36 (0.34)	0.04^c^ (0.02)	−0.65^c^ (0.27)	0.02 (0.02)
LAME	0.01 (0.22)	0.04^c^ (0.02)	−0.12 (0.22)	−0.01 (0.02)	−0.37 (0.24)	0.00 (0.02)
META	0.21 (0.22)	0.04^c^ (0.02)	−0.21 (0.24)	0.02 (0.02)	−0.35 (0.22)	−0.02 (0.02)
MISC	−0.40 (0.31)	−0.01 (0.02)	−0.07 (0.30)	0.04^c^ (0.02)	−0.35 (0.31)	−0.04^c^ (0.02)
THC	−0.05 (0.17)	0.04 (0.03)	−0.23 (0.18)	0.05^c^ (0.02)	−0.60^c^ (0.16)	−0.04^c^ (0.02)
Metritis	0.33 (0.48)	0.01 (0.02)	−0.21 (0.44)	0.02 (0.02)	−0.74 (0.39)	0.01 (0.02)
Retained placenta	0.19 (0.22)	0.04^c^ (0.02)	−0.27 (0.22)	0.02 (0.02)	−0.25 (0.23)	0.05^c^ (0.02)
Displaced abomasum	0.30 (0.21)	0.04^c^ (0.02)	−0.07 (0.23)	0.01 (0.02)	−0.27 (0.23)	−0.01 (0.02)
Ketosis	0.21 (0.20)	0.03 (0.02)	−0.07 (0.21)	0.04^c^ (0.02)	−0.43^c^ (0.20)	−0.02 (0.02)

^a^ Higher scores were assigned to taller cows for stature, more angular cows for dairy form, and more shallow cows for udder.

depth.

^b^ MAST, mastitis; REPRO, reproduction; LAME, lameness; META, metabolic, and MISC, miscellaneous.

^c^ Estimate was significantly different from zero based on 95% CI.

The phenotypic correlations between both stature and dairy form with health treatment costs for each of the 5 categories were small (Table 8); however, some were significantly different from zero. Small phenotypic correlations between the conformation traits and health disorders were also found by Lund et al. (1994) and Van Dorp et al. (1998). For stature, taller cows had greater cost of MAST (*r* = 0.04), LAME (*r* = 0.04), and META (*r* = 0.04). Furthermore, tall cows were associated (0.04) with increased displaced abomasum. A long-term selection study on body size of Holsteins (Becker et al., 2012) reported cows in a large body size line had 2.6 times the incidence of displaced abomasum as cows in a small body size line in first parity and more than 4 times the incidence of small-line cows in second parity. The large body size line had double the treatment cost for displaced abomasum compared with the small body size line (Becker et al., 2012).

Phenotypically, cows with more dairy form and, therefore, lower BCS (Dechow et al., 2003), were associated with higher costs of REPRO (*r* = 0.04) and also greater THC (*r* = 0.05) in the current study. Others (Hansen et al., 2002; Dechow et al., 2004) have hypothesized the mechanism of causation lies in the association of high dairy form with negative energy balance that is typically observed in pariparturient Holstein cows. Negative energy balance causes a depressed immune system (Goff and Horst, 1997), which could lead to more cost for health treatments—especially for postpartum metabolic disorders but also for some infectious diseases. Body condition score may be used as a predictor of the metabolic status of dairy cattle (Buonaiuto et al., 2022), and higher BCS may lower health treatment costs. However, the BCS of Holstein cows was not collected in the current study.

The genetic correlation (−0.60) of udder depth with THC indicated bulls transmitting shallower udders also transmitted lower cost of health treatments (Table 8). Perhaps, this genetic relationship was because cost of MAST had the largest genetic correlation (−0.84) among health treatment categories with udder depth. Rupp and Boichard (1999) reported a smaller genetic correlation (−0.26) between udder depth and incidence of mastitis. Furthermore, udder depth had a significant genetic correlation (−0.65) with REPRO cost. The favorable genetic relationships of udder depth with lower cost of MAST, REPRO, and THC may result from concurrent genetic selection for udder depth, SCS, and fertility. The phenotypic correlation (−0.11) between udder depth and MAST cost indicated cows with shallower udders had lower MAST cost. Udders closer to the ground may have functional problems while milking or may have more contact with bedding in stalls (Hansen et al., 1999).

### 3.5 Heritability of THC and production traits in multiple parities

The estimated heritability (0.25) of THC in first parity from the multivariate analysis (Table 9) was similar to the heritability (0.27) from the univariate analysis; however, both estimates were remarkably high compared to previous estimates of heritability for health traits. The estimates of heritability for THC in second (0.16) and third (0.17) parity were more modest than the result in first parity. Explanations for the decrease in heritability of THC with increasing parity may be the reduced number of cows contributing to the estimates and the culling of cows with greater THC from parity to parity. Cows with lower first-parity THC are more likely to remain in second and third parity. Zwald et al. (2004a) also reported higher heritability of health traits in first-parity cows than in multiparous cows, and they attributed this result to decreased genetic variance (or increased residual variance) from environmental factors such as poor management during the previous dry period. The results suggest substantial genetic control for THC in all 3 parities and, because of the negative impact of THC on cow profitability, selection against THC should provide substantial economic gain for dairy producers.

**TABLE 9:** Estimates of heritability (SE in parentheses) for total health treatment cost (THC) and 305-d production and SCS in parities 1 to 3 from multivariate analysis across parities.

	Parity		
Trait	1	2	3
THC	0.25^a^ (0.07)	0.16^a^ (0.06)	0.17 (0.11)
Milk	0.23^a^ (0.06)	0.20^a^ (0.06)	0.19^a^ (0.09)
Fat	0.20^a^ (0.06)	0.21^a^ (0.07)	0.14 (0.08)
Protein	0.20^a^ (0.06)	0.11^a^ (0.05)	0.12 (0.08)
Fat + protein	0.18^a^ (0.06)	0.16^a^ (0.06)	0.13 (0.08)
SCS	0.18^a^ (0.06)	0.19^a^ (0.07)	0.10 (0.09)

^a^ Estimate was significantly different from zero based on 95% CI.

The high estimated heritability (>0.25) for THC in the current study, may be because of dedicated health recording of data from these high-performance dairies. Health data from dairy herds are often inconsistently recorded and may not be complete and require additional labor to collect high quality phenotype health data (Hardie et al., 2023). Therefore, results of THC observed in the current study may be the result of accurate data recording by these dairy herds.

Some cows within farms had low THC per cow; however, other cows withing herds had high THC costs, and this variance may contribute to the total phenotypic variability. Some of the dairy herds in the study monitored cows more closely and thoroughly, and accrued more labor and possibility detected more health disorders of cows. Considerable variation existed for THC of cows in the eight herds; however, health costs of cows contributed to high costs of production withing these dairy herds. The costs of care, pharmaceuticals, and labor are difficult to determine for specific health disorders of cows, and therefore, phenotypic variation of cows exists within and across dairy herds.

Estimates of heritability for the production traits (Table 9) were similar to those used for routine genetic evaluation of US Holstein cows (VanRaden and Cole, 2014) for the production traits (h2 = 0.20) and for SCS (h2 = 0.12). However, estimates of heritability for 305-d production in first parity from this study were lower than the estimates reported by Rupp and Boichard (1999) of 0.26, 0.31, and 0.26 for milk, fat, and protein production, respectively, in French Holstein cows. The heritability (0.18) for SCS in this study is in agreement with the estimate of heritability (0.17) of Van Dorp et al. (1998).

Moderate heritabilities for production traits of dairy cattle have permitted substantial improvement in production over the past 50 years. The heritability of THC in this study suggests substantial improvement should likewise be possible for reduced health treatment cost of dairy cows if health treatments are recorded in a uniform manner on farms for the most common and most expensive health disorders.

**FIGURE 1:**
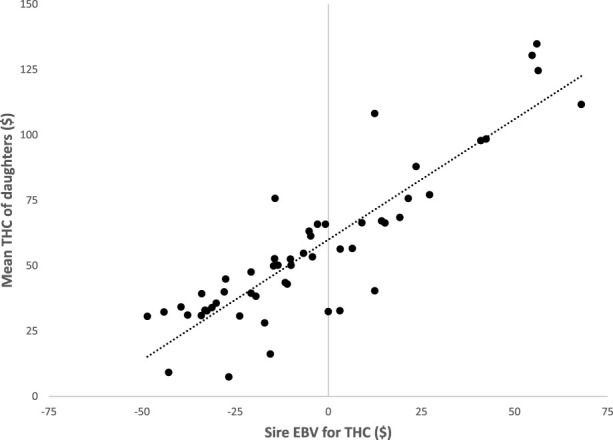
The EBV for total health cost (THC) of 53 sires with at least 10 daughters versus the mean THC of the corresponding daughters in first parity.

The EBV for THC of sires (*n* = 53) with at least 10 daughters from the univariate analysis of first-parity cows are plotted versus the mean THC of the corresponding daughters (Figure 1). The EBV for THC ranged from $67 to −$49, and this is a difference of roughly $116 between the highest and lowest in first parity. However, most of the sires had EBV for THC between $20 and −$40. The regression coefficient was 0.92, which indicated the EBV for THC of sires were very good predictors of the extent of THC for their daughters. The range of EBV for THC suggests sire selection could be highly effective in successfully reducing the THC of dairy cows. A limitation of this study may be results are from cows managed only in high-performance dairy herds in the upper Midwest of the United States. Results may be different for cows provided lower management levels or located in other environments, globally. Future research of analysis of THC would be to determine costs on a lifetime basis. The THC during lifetimes of cows is likely more impactful than cost based on lactational data, because lactational THC is subdivided based on calving events. The potential health complications during and shortly after calving have most impact on health cost however, cows that have high cost early in life likely have shortened longevity. Therefore, lifetime THC regressed on total days of herdlife and/or an analysis of THC per day in the herd may be more appropriate. Either of these analyses would likely favor cows with abnormally long lactations because cows with fewer calvings and longer lifespans should have less THC. For all of these reasons, lifetime THC needs to be interpreted within the context of total profitability, because individual cows with increased THC may have high productivity and long herdlife; likewise, cows with decreased THC may be low-producing or leave herds for other reasons. Cows that require less healthcare are also preferable from an animal welfare point of view.

## 4 Conclusion

The genetic evaluation of health traits for dairy cows in the United States has been inhibited by inconsistent and incomplete health data because producers in the United States are not incentivized or required to record health events. Most previous efforts to estimate genetic parameters of health traits have treatments limited to the recording of a single binary outcome per disorder in each parity. The comprehensive recording of health data by the 8 herds in this study permitted the determination of health treatment costs for 14 different types of treatment, but also enabled inclusion of multiple treatments per lactation for genetic analysis. These two factors provide more expression of genetic variation and may furnish a more appropriate data structure than binary data for a genetic analysis of health disorders.

Genetic correlations between THC and health treatment cost for each of 5 treatment categories were large and positive in first parity. The moderate genetic correlation between THC and 305-d milk production in first parity suggested historical selection for increased fluid milk production may have caused a correlated increase of THC in modern Holstein cows; however, our results suggest selection for fat (kg) and protein (kg) has a reduced association with THC. The high genetic correlation indicates selection for SCS can dramatically reduce THC in these herds.

Results from this study suggest the collection of uniform and comprehensive health treatment data in the United States is feasible using current herd management software. Perhaps herds may use herd health data for genetic selection of cows andfind effective utilization of treatment records and costs for herd management. Selection for reduced THC should also lessen the chances of antibiotic residues in meat and milk. Furthermore, genetic improvement of the health of dairy cows would lead to enhanced welfare of cows and an improved public perception of the dairy industry.

## Data Availability

The raw data supporting the conclusion of this article will be made available by the authors, without undue reservation.
